# Large-surface-area BN nanosheets and their utilization in polymeric composites with improved thermal and dielectric properties

**DOI:** 10.1186/1556-276X-7-662

**Published:** 2012-11-30

**Authors:** Xuebin Wang, Amir Pakdel, Jun Zhang, Qunhong Weng, Tianyou Zhai, Chunyi Zhi, Dmitri Golberg, Yoshio Bando

**Affiliations:** 1 International Center for Materials Nanoarchitectonics (WPI-MANA), National Institute for Materials Science (NIMS), Namiki 1-1, Tsukuba, Ibaraki 305-0044, Japan; 2 Department of Nano-Science and Nano-Engineering, Faculty of Science and Engineering, Waseda University, Okubo 3-4-1, Shinjuku-ku, Tokyo, 169-8555, Japan; 3 Department of Physics and Materials Science, City University of Hong Kong, Tat Chee Avenue, Kowloon, Hong Kong 999077, China

**Keywords:** BN nanosheet, Polymeric composite, Thermal conductivity, Dielectric constant

## Abstract

High-throughput few-layered BN nanosheets have been synthesized through a facile chemical blowing route. They possess large lateral dimensions and high surface area, which are beneficial to fabricate effectively reinforced polymeric composites. The demonstrated composites made of polymethyl methacrylate and BN nanosheets revealed excellent thermal stability, 2.5-fold improved dielectric constant, and 17-fold enhanced thermal conductivity. The results indicate multifunctional practical applications of such polymeric composites in many specific fields, such as thermoconductive insulating long-lifetime packaging for electrical circuits.

## Background

Honeycomb-like mono-/few-layered hexagonal boron nitride (*h*-BN; also called ‘white’ graphene) is a structural analogue of graphene, which may serve as one of the outstanding representatives of 2D crystals due to its unique physics and diverse functionalities [1,2]. The robust B-N bonding within a BN layer, even stronger than C-C bonding in graphene, makes mono-/few-layered BN nanosheets highly thermoconductive (*ca.* thermal conductivity of 100 to 1,000 W/mK), mechanically strong and elastic, and thermally and chemically stable [3,4]. Partially ionic B-N bonds, different from pure covalent bonds in graphene, make BN nanosheets an intrinsic insulator with a wide band gap (*ca.* 5.5 eV) valuable for dielectric applications and deep ultraviolet luminescence. Besides, the weak van der Waals bonds out of planes of few-layered BN nanosheets are advantageous for good solid-state lubricants.

Quick heat-releasing and good electrical insulation are required in the packaging materials in high-speed electronics, and polymer materials with a high dielectric constant are attractive for large capacitors and high-*k* gate in flexible electronics [5-7]. The standard polymer materials normally have low thermal conductivity. One approach toward preparing highly thermal conductive polymeric materials is to embed fillers with high thermal conductivity, such as traditional silicon nitride, aluminum nitride, and boron nitride microparticles. Nanomaterials are more effective fillers for the so-called nanocomposites due to their developed surfaces and high aspect ratios. The polymers embedded with graphenes or other conductive fillers may exhibit high thermal conductivities and dielectric constants before the percolation threshold [8]; however, the possible electrical current leakage is undesired. BN materials exhibit good electrical insulation to deal with these drawbacks. Comparing with 0D BN nanoparticles [9] and 1D BN nanofibers/tubes [10-14], 2D BN nanosheets maximally expose their basal (002) crystal planes; therefore, the regarded excellent in-plane properties become dominant because both directions parallel to the (002) planes substantially work for phonon transport in a BN sheet. Intrinsic thermal conductivity of BN nanosheets is notably higher than the reported values of AlN powders and BN powders/nanotubes [15]. BN nanosheets are thus envisaged to be one of the best fillers in composites owing to the highly insulating and thermoconductive properties.

Filling of BN nanosheets into polymeric or ceramic composites requires a sufficient mass of BN nanosheets. The current methods, such as mechanical cleavage, solution exfoliation [16-18], high-energy electron beam irradiation, reaction of boric acid and urea [19], unwrapping nanotubes [2], and chemical vapor deposition [20-24], have been utilized to successfully fabricate BN nanosheets; however, mass quantities of BN nanosheets via those methods are still not available on the market due to many problems in reliable chemical intercalations and exfoliations. Recently, a new strategy, the so-called ‘chemical blowing’, has been developed by us [25,26], which relies on making large bubbles with atomically thin B-N-H polymer walls starting from a precursor ammonia borane compound (AB, which is relatively cheap) and then annealing polymer bubbles into BN ones having ultra-thin BN walls, i.e., BN nanosheets. Here, we use the regarded chemical blowing technology to prepare large amount of BN nanosheets (gram-level) and reveal their high surface area. Based on the high throughput, high surface area, and unique 2D-crystal properties, the produced BN nanosheets effectively perform as excellent fillers in polymeric composites for improving thermal stability, thermal conductivity, and dielectric properties.

## Methods

Commercial fresh AB (Sigma-Aldrich Corporation, St. Louis, USA) was first pre-treated at 80°C for 1 h. Using a 8°C/min heating rate, the precursors were heated to 1,300°C to obtain the BN products. The products were characterized by scanning electron microscopy (SEM, Hitachi S-4800, Tokyo, Japan), high-resolution transmission electron microscopy (HRTEM, JEOL JEM-3000F, Tokyo, Japan), atomic force microscopy (AFM, JEOL JSPM-5200), electron energy loss spectroscopy (EELS, attachment in TEM), and nitrogen adsorption-desorption measurements carried out at liquid nitrogen temperature (Quantachrome Autosorb-1, Boynton Beach, FL, USA). Brunauer-Emmett-Teller (BET) surface area was estimated over a relative pressure range of 0.05 to 0.3 P/P_0_. Pore distributions were analyzed using a Barrett-Joyner-Halenda method.

To fabricate polymer/BN composites, as-grown BN products were dispersed in a polymethyl methacrylate (PMMA)/chloroform solution with a controlled weight ratio. The mixture was then spread on a glass plate; this made the solvent naturally volatilized. The residual solvent was removed by further drying at 50°C. The composites were studied by thermogravimetry (TG; Rigaku Thermo plus TG 8120, Tokyo, Japan) and differential scanning calorimetry (DSC; Rigaku Thermo plus EVO DSC8230), thermoconductivity analysis (thermal constant analyzer of Kyoto Electronics Manufacturing Co., Ltd., Kyoto, Japan) following a hot disk method, and dielectric constant analysis (Wayne Kerr precision component analyzer, West Sussex, UK).

## Results and discussion

**Figure 1:**
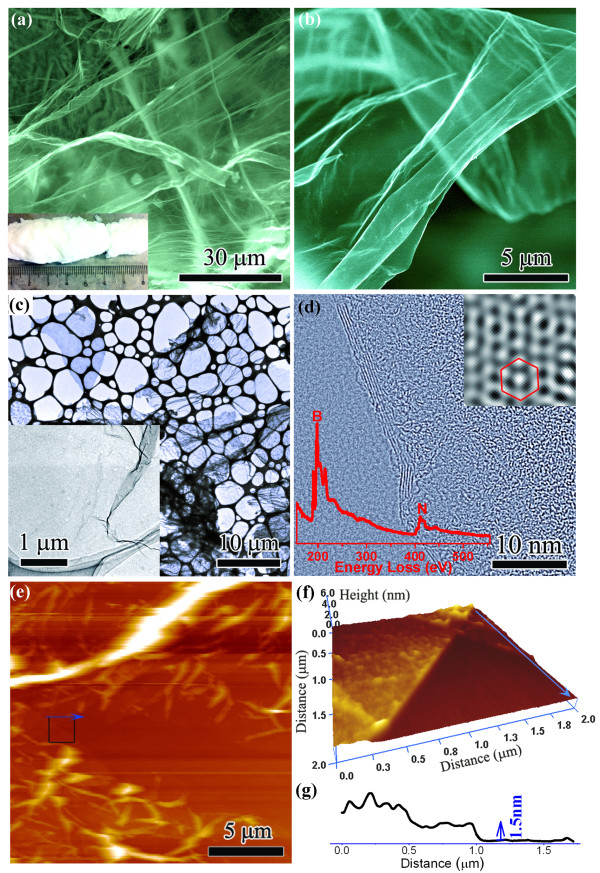
**Characterization of BN nanosheets.** (**a**) SEM image of laterally large BN nanosheets. The inset is a BN product of around 100 mg in weight obtained during a single experimental run. (**b**) A magnified image of an ultra-thin BN nanosheet. (**c**) TEM image of plentiful BN nanosheets which were dispersed in ethanol. The inset is a selected magnified image showing a detailed folded corrugation landscape. (**d**) HRTEM image of a six-layered BN nanosheet. The inset image is a zoomed-in picture indicating a perfect honeycomb-like BN crystal lattice. The inset profile is the corresponding EELS of BN nanosheets. (**e**) AFM tapping mode image of two pieces of large BN nanosheets. (**f**) 3D view of the marked region in (**e**). (**g**) Cross-sectional profile along the scan marked in (**e**) and (**f**).

The synthesized BN product is a sponge-like light solid with a density of 6 mg/cm^3^ (inset of Figure 1a). It consists of large bubble-like structures with a diameter of tens of micrometers. The walls of the bubbles are spacious BN few-layered nanosheets, up to 100 μm in lateral size, as shown in SEM images (Figure 1a,b). There are many surface corrugations on a sub-micrometer scale, as additionally confirmed in a TEM image (Figure 1c). The cavity within a corrugation is estimated to be tens of nanometers in size, as indicated in the inset of Figure 1c. A six-atomic layered BN nanosheet with clear folded edges is shown in Figure 1d. The interlayer spacing is 0.342 nm, slightly larger than in bulk BN, probably owing to surface atom relaxation. The surface of nanosheets is not so flat and prone to many fluctuations and ripples on the nanometer-level scales. These may result from the thermodynamical relaxation within the thin films. The honeycomb-like hexagonal lattice (the inset of Figure 1d) illuminates a high crystal quality. The perfect BN stoichiometry was documented by EELS (the inset of Figure 1d). The possible oxygen and excessive hydrogen elements were removed under high-temperature crystallization to yield stoichiometric BN due to volatile boron-oxygen and nitrogen-hydrogen species. The large-area ultra-thin BN nanosheets are additionally confirmed in an AFM image (Figure 1e,f). A thin nanosheet with a thickness of 1.5 nm that corresponds to *ca.* three to four BN layers is shown in Figure 1f,g.

**Figure 2:**
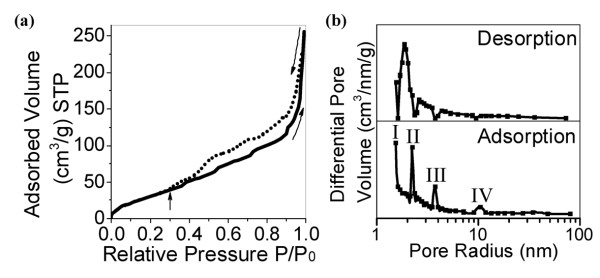
**Surface area and pore analysis of BN nanosheets.** (**a**) Nitrogen adsorption and desorption isotherms of as-grown BN nanosheets. (**b**) Corresponding pore radius distribution curves.

The nitrogen adsorption-desorption isotherms of BN nanosheets show a type IV isotherm characterized by a hysteresis and steep slope (Figure 2a). The unsaturated adsorption at the highest pressure part reflects macropores and secondary piled pores. The stepped condensations I to IV, i.e., ones occurring at 3, 4, 8, and 20 nm, respectively (Figure 2b), result from the peculiar structures on the sheet surface for different geometry scales, such as surface atomic defects, ripples, and corrugations. The entire hysteresis loop resembles the type H3 one based on the classification of the International Union of Pure and Applied Chemistry, which is a character of open-shaped capillaries between the parallel layers. The delayed desorption ranged from 1.0 to 0.3 results from the piled slits in a corrugated landscape or between agglomerated sheets. These slit-like cavities dominate the desorption dynamics, giving a quickest desorption around 4 nm (Figure 2b). The evaporation of liquid N_2_ from the surface-attached structures, such as ripples, is combined with that from the slit-like cavities, so that the reverse steep evaporations corresponding to forward steep condensations are absent. The as-grown BN products possess large specific surface area (SSA), 140 m^2^/g based on a BET method, which is larger than 50 m^2^/g of porous BN or 27 m^2^/g of BN nanoparticles [9,27]. The SSA resulted from the mesopores and macropores, while there is no contribution from micropores according to the *t*-plot analysis. This configuration is beneficial to support high-temperature catalysts, and to create more interfaces between BN and polymer molecules in polymeric composites, because the too-small pores cannot be accessed by polymer molecules to create links and effective BN-polymer interactions. The specific volume is as high as 0.40 cm^3^/g. To evidence the macroporous structures of the as-grown BN, we milled and crushed the BN product into small fragments of BN nanosheets. The fragments may easily agglomerate and re-stack, which makes the part of surfaces and surface-attached cavities embedded inside and inaccessible for N_2_ molecules. Hence, the SSA of milled BN decreases to only 100 m^2^/g. This confirms the advantages of the interconnected structure which prevents severe agglomeration and maximally exposes the surface of BN sheets.

**Figure 3:**
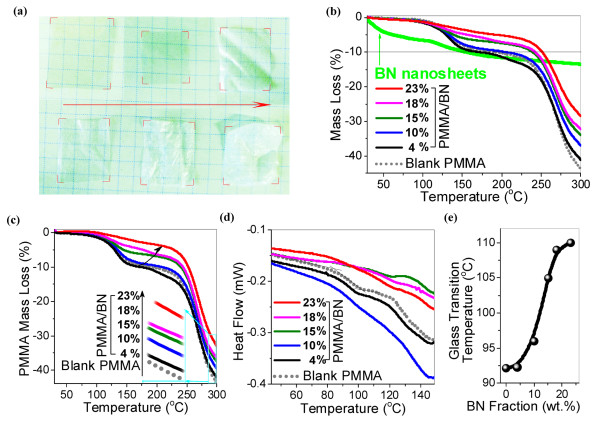
**Thermal stability of polymeric composites.** (**a**) Optical photos of blank PMMA and PMMA composites with 4, 10, 15, 18 and 23 wt.% of BN arranged from left to right and from top to bottom. (**b**) TG curves of blank PMMA, PMMA/BN composites, and pure BN nanosheets. The mass loss of BN nanosheets might result from the loss of surface-adsorbed/functionalized water or organic groups due to ionic B-N bond characteristics. (**c**) Weight change of PMMA components in blank PMMA and in PMMA/BN composites. The mass loss was normalized to the weight of PMMA after removal of the BN component mass loss. (**d**) Typical DSC curves of blank PMMA and PMMA/BN composites. Second scans were used here to release a thermal stress. *T*_g_ was determined at the mid-point in a three-tangent method. (**e**) The increased *T*_g_ along with increasing filling fraction of BN nanosheets.

The produced large-area 2D BN nanosheets can fully demonstrate the unique properties of (002) *sp*^2^ hybridized crystal planes. Their large SSA further prolongs interactional interfaces and intensifies interfacial interactions between BN and polymer molecules, and thus forms diverse functionally reinforced plastics. Herein, we fabricated the PMMA/BN composites with different BN fractions. The composites kept around half of transparency until 10 wt.% of BN sheets (Figure 3a). The thermal stability of PMMA/BN composites was characterized by TG tests. The composites showed increased thermal stability that increased along with increasing BN fractions (Figure 3b). The onset degradation temperatures of the composites (temperature at 10% mass loss) are higher than that of blank PMMA except a slightly lower onset temperature for 4 wt.% PMMA/BN. The mass loss was then normalized into the weight of only PMMA components to clearly explicate the thermal stability of PMMA in composites (Figure 3c). The two-step degradation of PMMA includes an initiation of unzipping vinylidene end groups and further random scission of polymer links [28]. The surface groups of BN such as oxidative B-O may react with the polymer end radicals. This reaction can suppress unzipping of the polymer chain and stabilize the composite system [29]. This interfacial-stabilizing effect was amplified for high SSA of BN fillers, so that the mass loss at 120°C to 200°C quickly becomes weaker for PMMA mixed with BN (except 4 wt.% PMMA/BN), and the onset temperatures of high-filling-fraction PMMA/BN composites are much higher than that of a blank PMMA. The PMMA molecules which were attached on BN surfaces are so stable that they can be kept even at a high temperature, and this leads to the increased residual at 300°C (the inset of Figure 3c). For a low fraction of BN in PMMA (4 wt.%), the thermal behavior of PMMA is slightly more active than that of the blank PMMA below 250°C, which may result from another unclear active radical interfacial reaction. The thermal stability was also checked by glass transition using DSC. The end of glass transition clearly increases along with an increase in the BN filling fraction (Figure 3d). Quantitatively, the glass transition temperature (*T*_g_) was increased from 92°C for the blank PMMA via 105°C for PMMA with 15 wt.% of BN to 110°C for a 23 wt.% BN sample (Figure 3e). This indicates that thermal mobility of polymer chains is affected due to strong and confined BN-PMMA interfacial interactions. The confined PMMA chains would exhibit slower dynamics and, thus, promote an increase in glass transition temperature.

**Figure 4:**
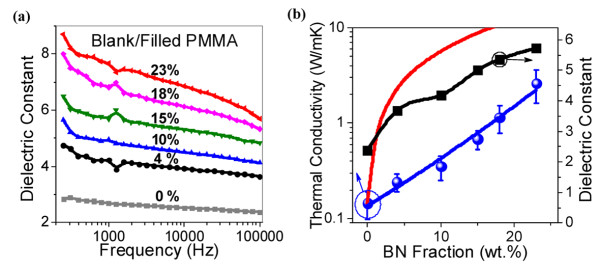
**Dielectric and thermal conductive properties of polymeric composites.** (**a**) Frequency-dependent dielectric constants of blank PMMA and PMMA/BN composites. (**b**) Increased dielectric constant (taken at 10^6^ Hz) and thermal conductivity with increasing BN fraction in PMMA/BN composites. Blue curve is the fitting to experimental points using the Agari model; red one is the upper limit of thermal conductivities in theory from a parallel model.

The dielectric constants of PMMA/BN composites were improved, as shown in Figure 4a. Dielectric constants increase with increasing BN fraction. A maximum reinforcement for PMMA with 23 wt.% BN is 2.5 times of blank PMMA at 10^6^ Hz. The measured dielectric constants were higher than the theoretically calculated mixing average values based on logarithmic mixing rule or Maxwell-Garnett approximation [30]. These exceptionally high dielectric constants should mainly result from special interfaces. Large-SSA BN nanosheets cause abundant inter-phase interfaces, which increase the interfacial polarization. Secondly, lots of surface states on polycrystalline BN nanosheets, such as vacancies and hydroxides, may induce additional ionic and electronic relaxation polarizations. These polarizations contribute with a large dielectric constant and also lead to an increased dielectric loss, which should appear around 10^6^ Hz (as confirmed in the experiments). Dielectric properties of materials also depend on the nature of the electrical contact during measurements [31]. The surface roughness of polymeric composites increases with BN filling fraction, which may partially contribute to the exceptional results. The obtained polymer composites with a high dielectric constant are attractive for potential high-charge storage capacitors and artificial muscles.

In addition to be a reliable dielectric material, PMMA/BN composites also demonstrate remarkable thermal conductivity (Figure 4b). The highest thermal conductivity is 2.6 W/mK for a PMMA with 23 wt.% BN, thus displaying a 17-fold increase with respect to the blank PMMA. The increase in thermal conductivity is higher than that of the previous polymeric composites that used fillers of BN microparticles [32] and nanotubes [33] at the same filling fractions. The relationship between thermal conductivity of composites and BN filling fraction may be fitted by the model of Agari et al. as follows [34]:

(1) $$\text{log}\lambda_{\text{c}}=\phi C_{2}\text{log}\lambda_{\text{f}}+\left(1-\phi \right)C_{1}\text{log}\lambda_{\text{m}}\text{,}$$

where *λ*_c_, *λ*_f_, and *λ*_m_ are the thermal conductivities of composites, BN fillers, and PMMA matrices, respectively; *ϕ* is the volume fraction of BN fillers. Parameter *C*_1_ relates to structures of polymer matrix; and *C*_2_ means the difficulty level in forming conductive chains of fillers. In this fitting, *C*_1_ = 0.94; *C*_2_ = 3.9. The *C*_2_ is generally larger than that of powder fillers, implying an easily constructed thermal conductive path, which results from the large lateral size of BN nanosheets grown using chemical blowing technology (tens of μm lateral size of BN nanosheets dispersed in a solution). Together with the abundant interfaces and strong interfacial interactions, the heat in a polymer matrix can, thus, be easily collected and conducted by BN fillers, resulting in a high thermoconductive of the composites.

## Conclusions

To sum up, mass production of meso-/macro-porous large-SSA few-layered BN nanosheets is realized; the sheets have successfully been integrated into PMMA polymeric composites. The outstanding (002)-crystal plane properties and abundant interfaces of BN nanosheets are utilized to increase the thermal stability, thermal conductivity, and dielectric properties of the composites, i.e., 17-fold gained thermal conductivity, 2.5-fold increased dielectric constant, and a 18°C increased glass transition temperature were documented. The fabricated PMMA/BN composite plastics are, thus, envisaged to be valuable for diverse functional applications in many fields, especially for the new-generation thermoconductive insulating long-lifetime packaging materials.

## Competing interests

The authors declare that they have no competing interests.

## Authors' contributions

XW carried out the synthesis and characterization of BN nanosheets, and made the PMMA-BN composites. AP, JZ, QW, TZ, and CZ assisted to operate the horizontal furnace for the synthesis of the nanosheets. XW and CZ conducted the analysis on thermal conductivity and dielectric constants of composites. YB, DG, and CZ supervised the project. XW and DG wrote the manuscript with referring other authors' comments. All authors read and approved the final manuscript.

## Authors' information

XW is a junior researcher of MANA, NIMS, as well as a Ph.D. candidate of Waseda University (Japan) under the supervision of Prof. YB. He obtained his bachelor and master degrees at Nanjing University. At present, he focuses on the syntheses and applications of 2D *sp*^2^ hybrid crystals, such as BN nanosheets and graphenes. CZ is a scientist of MANA, NIMS, and is moving to City University of Hong Kong (China) as an assistant professor. He is an expert in the fields of inorganic nanomaterials and functional polymeric composites. DG and YB are two group leaders and professors in MANA, NIMS.
